# Bacterial Sinusitis in the COVID-19 Era: A Reminder for Antibiotic Stewardship

**DOI:** 10.7759/cureus.19453

**Published:** 2021-11-10

**Authors:** Manasa Brown, Shivanjali Shankaran

**Affiliations:** 1 Infectious Disease, Rush University Medical Center, Chicago, USA

**Keywords:** multidrug resistance, steroid use, antibiotic stewardship, bacterial sinusitis, covid-19

## Abstract

The COVID-19 pandemic has necessitated the trial of novel treatment regimens to improve clinical outcomes. However, the liberal use of antibiotic and steroid therapy during this period may have also contributed to unintended consequences including the development of multidrug-resistant (MDR) bacterial infections. In this report, we discuss the case of a 76-year-old woman treated with an extended course of steroids for COVID-19 infection. The patient then developed MDR bacterial sinusitis requiring multiple courses of antibiotics complicated by medication side effects. Thus, this case highlights the continued importance of discretion in long-term steroid use and antibiotic stewardship.

## Introduction

Sinusitis is one of the most prevalent conditions prompting clinic visits in the United States, accounting for up to $2.4 billion yearly in medical costs [1]. While viral upper respiratory tract syndromes typically subside within one week, bacterial sinusitis can persist for up to 10 days or longer leading to possible additional complications [2]. Bacterial sinusitis typically manifests with purulent nasal drainage associated with nasal obstruction and facial pain [2]. Risk factors include immunodeficiency from prolonged steroid use, human immunodeficiency virus, or diabetes mellitus as well as anatomic defects including recent trauma or facial fractures [1]. Increased steroid and antibiotic use in the COVID-19 era may predispose individuals to develop multidrug-resistant (MDR) bacterial infections [3]. While mucor sinusitis has been increasingly described as a complication of prolonged steroid use in the treatment of COVID-19, bacterial sinusitis has not been described in this same setting [4]. Here, we present a case of bacterial sinusitis and subsequent complications in an individual treated with prolonged steroids and antibiotics for COVID-19 infection.

## Case presentation

**Figure 1:**
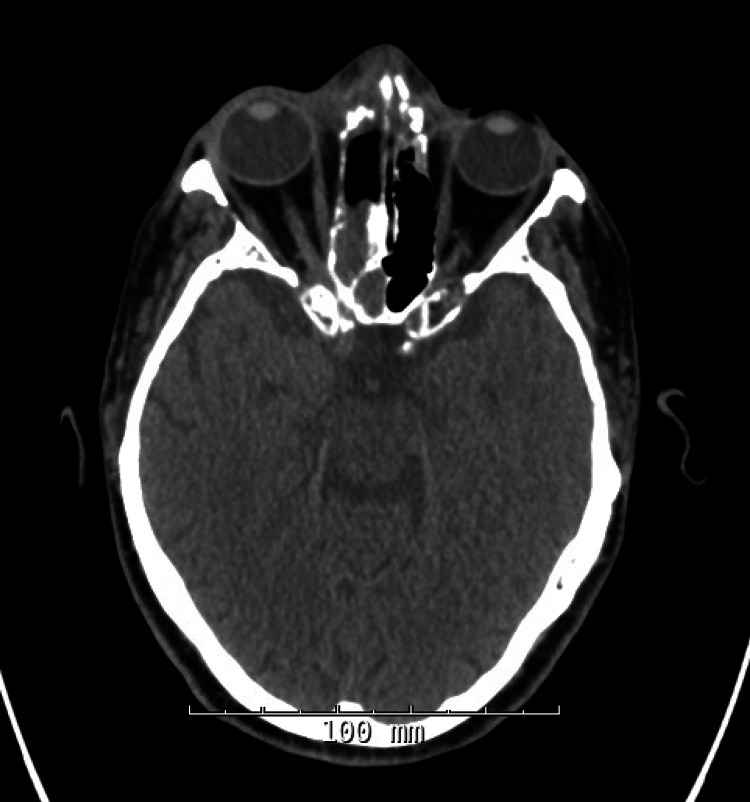
CT Scan With Multifocal Sinusitis

**Figure 2:**
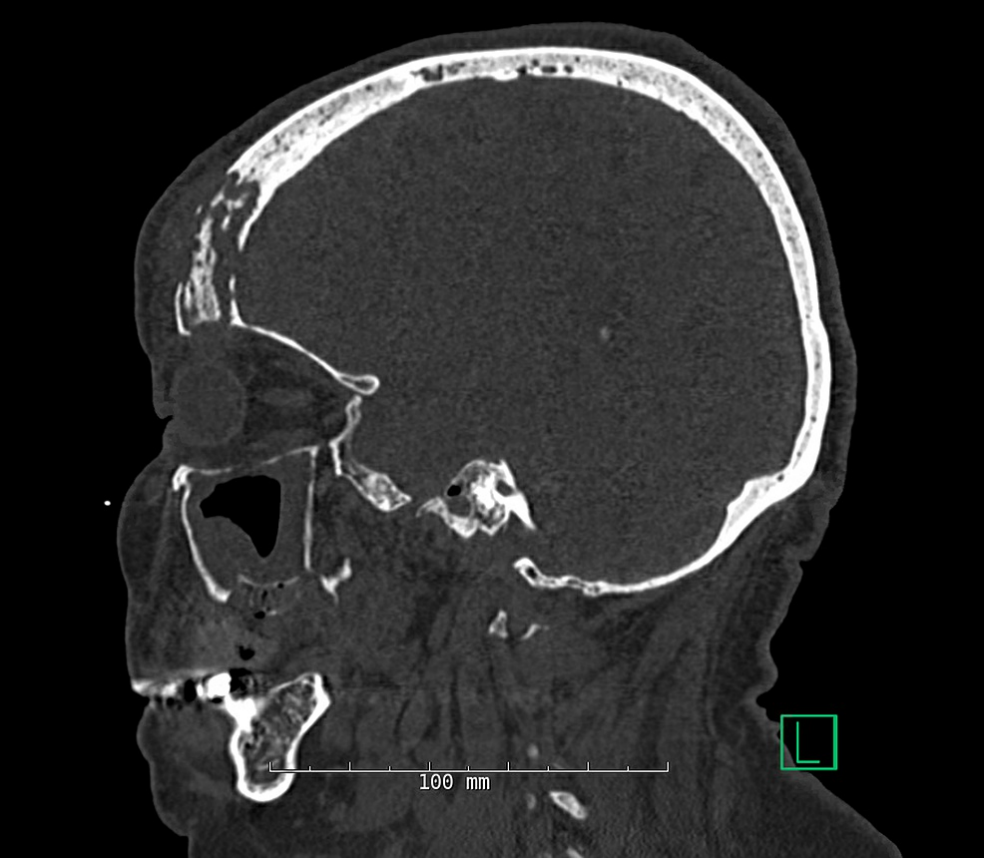
CT Head With Dehiscence of Inner Table

A 76-year-old female with a past medical history of hypertension presented to the emergency department with persistent forehead swelling two months after recovering from COVID-19 infection. The patient was diagnosed with COVID-19 pneumonia in Egypt and completed a six-week course of dexamethasone. Per family, she also received multiple antibiotics during this time, the names and courses of which were unknown. She subsequently developed progressively tender frontal scalp swelling. Initial evaluation with otorhinolaryngology (ENT) in Egypt showed mucor-like organisms on pathology. She then came to the United States and was admitted for further evaluation. Her CT head showed evidence of multifocal sinusitis with breach of the inner and outer tables of the right frontal sinus with dural enhancement (Figure 1). The CT scan also confirmed dehiscence of the inner table (Figure 2). She was then empirically started on a course of Amphotericin for post-COVID mucormycosis. The patient underwent surgical debridement of significant underlying purulence with intraoperative cultures growing extended-spectrum beta-lactamase-producing *Escherichia coli* and methicillin-resistant *Staphylococcus aureus*. Due to these culture results, the patient was initiated on vancomycin and meropenem with minimal improvement in edema. She underwent repeat surgical debridement two weeks later with cultures redemonstrating previously identified bacteria with rare *Pseudomonas aeruginosa* growth as well. The patient endorsed moderate improvement in frontal scalp tenderness following this debridement. She was thus discharged home on a six-week course of IV vancomycin through a peripherally inserted central catheter, PO levofloxacin, and PO posaconazole, with a plan to continue the latter until fungal cultures were finalized. Two weeks later, she presented to the outpatient clinic with new-onset generalized weakness. Initial workup revealed profound hypokalemia (potassium of 1.5 mEq/L; normal range 3.4-5.3 mEq/L) and acute kidney injury (creatinine of 3.03 mg/dL from baseline of 1.20 mg/dL; normal range 0.65-1.00 mg/dL) likely due to vancomycin-related renal derangements and potassium wasting due to posaconazole. The patient was admitted for fluid resuscitation and switched from vancomycin to daptomycin upon discharge to prevent further renal impairment. As all her fungal cultures were negative and no fungal forms were seen on pathology, posaconazole was discontinued. The patient was able to complete her course of treatment, though it was complicated by mild creatine kinase elevation due to daptomycin.

**Figure 3:**
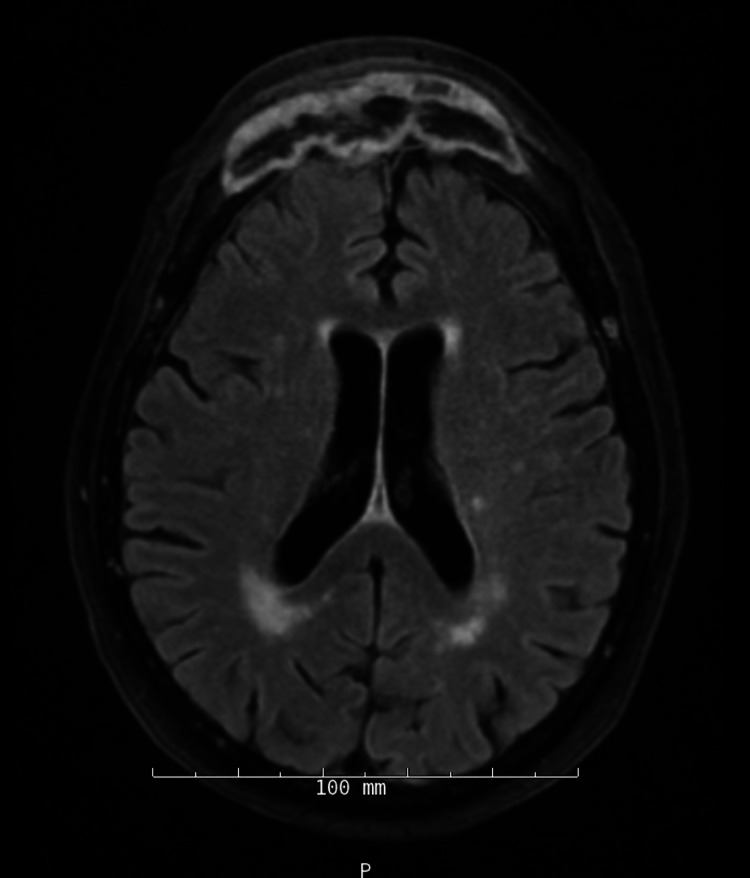
T2 Axial Flair MRI Brain With Persistent Frontal Sinusitis

Repeat MRI at the end of her antibiotic course demonstrated decreased soft tissue thickening along the anterior skull base and superior nasal cavity with improvement in mucosal thickening involving maxillary sinuses, sphenoid sinuses, and residual ethmoid air cells (Figure 3). However, two weeks later, the patient complained of persistent frontal tenderness and was noted to have new periorbital swelling. Due to frontal sinus sequestrum formation and worsening of symptoms soon after antibiotics were stopped, the patient was recommended to undergo craniectomy to achieve definitive source control. She also had to be restarted on daptomycin and meropenem while awaiting surgery.

## Discussion

As the COVID-19 pandemic has evolved, multimodal therapeutic interventions have been identified, with immunosuppressives such as steroids and interleukin 6 inhibitors being frequently used [5]. In addition, a significant percentage of patients receive broad-spectrum antibiotics during their course of treatment, particularly those admitted to the intensive care units, those with prolonged hospital stays, and those with superimposed bacterial infections such as ventilator-associated pneumonia [6,7]. This antibiotic use in the setting of other risk factors including personal protective equipment shortage and hospital overcrowding may have contributed to the development of MDR bacterial and fungal infections [8]. For instance, rhino-orbital mucormycosis has been well described as a consequence of treatment for COVID-19 infection, highlighting the impact of extensive steroid and broad-spectrum antibiotic use in promoting the development of opportunistic fungal infections [4]. However, to our knowledge, there have been no prior case reports describing the progression of subacute MDR bacterial sinusitis in a patient with prior COVID-19 pneumonia. In many patients, including ours, the presentation of bacterial sinusitis may resemble that of mucormycosis, given overlap in symptoms and risk factors, such as facial swelling and sinus involvement in the setting of immunosuppression or recent infection [9]. Given the differences in treatment course and the significant mortality and morbidity associated with both disease processes, accurate recognition and rapid management are critical in preventing further complications associated with mucormycosis and bacterial sinusitis. Clinicians should also be aware of the side effects associated with the use of antifungals and antibiotics, as demonstrated in our patient above. Limiting antibiotic use and duration in the management of COVID-19 infection, and the judicious use of both antibiotics and steroids may help decrease the risk of bacterial superinfection as well as colonization with MDR organisms.

## Conclusions

Here we describe a case of subacute pansinusitis caused by multiple MDR bacteria, leading to prolonged inpatient and outpatient treatment, significant antibiotic side effects, and the development of complications requiring extensive surgery to ensure cure. As such, this case highlights the importance of antibiotic stewardship and discretion in the use of broad-spectrum antibiotics, as well as steroids, in the treatment of COVID-19. Though mucor sinusitis has been more commonly described as a complication, clinicians should be aware of the possibility of a subacute bacterial process in patients presenting with sinusitis.
